# Batch Specular Plane Flatness Measurements Based on Phase Measuring Deflectometry

**DOI:** 10.3390/s24092693

**Published:** 2024-04-24

**Authors:** Zhuotong Li, Dongxue Wang, Lei Liu, Xiaodong Zhang

**Affiliations:** State Key Laboratory of Precision Measuring Technology & Instruments, Laboratory of MicroNano Manufacturing Technology, Tianjin University, Tianjin 300072, China; lzt_1721@tju.edu.cn (Z.L.); 2020202196@tju.edu.cn (D.W.); 3015202152@tju.edu.cn (L.L.)

**Keywords:** batch specular plane flatness, phase measuring deflectometry, discrete surface measurement, rapid reconstruction

## Abstract

Flatness is a critical parameter in the manufacturing industry, directly impacting the fit and overall product performance. As the efficiency of manufacturing continues to advance, there is an increasing demand for more accurate and efficient measurement techniques. Existing methods often struggle to strike a balance between precision and efficiency. In response, this article introduces a novel approach that is capable of achieving high-precision and rapid measurements concerning multiple surfaces. By enhancing the traditional phase measuring deflectometry (PMD) method, employing a matching technique based on polar lines and normal vector constraints to address discrete surface measurement challenges, and implementing a plane pre-positioning method to tackle low efficiency in binocular matching and solving, we successfully performed swift and synchronized measurements for a large batch of specular surfaces and obtained the three-dimensional surface profile of each measured surface. Through experimental validation, the method proposed in this paper can perform the batch measurement of specular planes while maintaining high measurement accuracy.

## 1. Introduction

Flatness is an important indicator in the industrial field and is directly related to the performance of many industrial components. For example, the flatness of the sealing face directly affects the sealing effect of the part [1]. The flatness of the wafer has a very important influence on the subsequent manufacturing process chain of integrated circuits [2]. And the flatness of the optical mirror directly affects image quality or beam quality [3]. It is evident that flatness plays a crucial role in the industrial application of components. Therefore, in both the production and quality control processes of parts, the measurement of flatness is indispensable. Furthermore, with the acceleration of industrial production, there is an increasing demand for more efficient measurement. If we can rapidly and simultaneously measure multiple planes in the measurement process, it will significantly enhance measurement efficiency, which holds high importance in research and application.

Commonly used methods for flatness measurement include coordinate-measuring machine [4] (CMM) and interferometry [5]. CMM employs a point-to-point measurement approach, which remains unaffected by the material of the parts under inspection and can assess surfaces with steep slopes. However, the point-to-point measurement method is notably slow and costly. On the other hand, interferometry is a full-field technique renowned for its high measurement accuracy, often utilized for standard component measurements. However, interferometric measurement systems come with a high cost; demand the precise leveling of the components under examination; and exhibit sensitivity to environmental factors, such as vibrations. These limitations hinder the enhancement of measurement efficiency and the widespread adoption of these techniques. As measurement technologies have advanced, phase measuring deflectometry (PMD) has been intensively studied as a measurement method applicable to reflective surfaces [6]. This method employs a surface-based measurement approach, providing a wide measurement field of view and high measurement precision without requiring precise adjustments to the surface being measured.

Dealing with the research challenges related to measurement efficiency, measurement accuracy, and the measurement of discrete surfaces in the PMD, many researchers have conducted in-depth studies on them. The single-camera measurement method based on the reference plane [7] can be used to measure specular near-plane surfaces, with fast reconstruction speeds. However, this method needs to ensure that the measured specular surface is very close to a reference with a known position, which limits the application scenarios of the measurement system. The binocular measurement system [8,9] determines the normal vectors of the measured surface by matching the normal vectors computed by the two cameras, which has high measurement accuracy, but the process of matching and searching between the binoculars is very time-consuming, which is not conducive to the improvement in inspection efficiency. A seed-point iterative surface reconstruction algorithm has been proposed to expedite binocular matching. This approach involves identifying a seed point in the binocular view and applying iterative principles from monocular deflectometry for reconstruction [10,11]. For fast iterative methods, both the localization of seed points and the study of iterative algorithms have been thoroughly researched [12,13]. The methods mentioned above cannot directly measure discrete surfaces, as they require the initial calculation of the surface’s normal vector, followed by surface shape integration and reconstruction. Liu et al. proposed a method for calculating the depth directly from the phase [14], called direct PMD (DPMD), which does not need to solve the normal vector, but its measurement system needs two screens and a beam splitter, and its measurement accuracy is not high. Considering the limitations of existing DPMD technologies, Wang et al. proposed a stereo-DPMD method to perform the high accuracy measurement of structured specular surfaces [15]. To minimize the interference of ambient light in measurements, Chang et al. refined the traditional structure and proposed a DPMD measurement method based on infrared light, but the system’s structure is complex [16]. In addition, Gao et al. proposed a system architecture for measuring structured specular surfaces (i.e., discontinuous specular surfaces and continuous non-differentiable specular surfaces). A novel near optical coaxial PMD was proposed by utilizing a plate beam splitter, thereby significantly reducing measurement shadows caused by discontinuous structures [17]. Additionally, they introduced an automatic segmentation technique based on gradient variation features and three-dimensional coordinate data of the measured structured specular surface [18]. However, this algorithm requires the initial computation of surface gradients. Zhang et al. [19] proposed the integration of slope information within each continuous surface area to enhance measurement accuracy. Subsequently, they calculated the absolute position of the height reference point for each continuous surface, enabling the evaluation of relative positions between different surfaces. However, this approach requires the measured surface to have corner features, and the algorithm’s complexity for binocular matching remains unaddressed.

In summary, while PMD is suitable for measuring the flatness of specular planes, there remains a gap in research regarding the rapid measurement of a large number of specular planes. This paper addresses this issue by establishing a binocular PMD system and optimizing the algorithm to achieve rapid and high-precision measurements of multiple planes. To address the dispersion among multiple planes, we introduce a method based on polar lines and normal vector constraints for matching corresponding planes. To address the drawback of extended search times in binocular systems, the search process for matching points is replaced with plane pre-positioning, resulting in a substantial enhancement in measurement efficiency, all while maintaining measurement accuracy. Ultimately, this approach enables the rapid and high-precision batch measurement of the three-dimensional profile of specular plane surfaces.

## 2. Methods

### 2.1. Measurement Fundamentals

**Figure 1:**
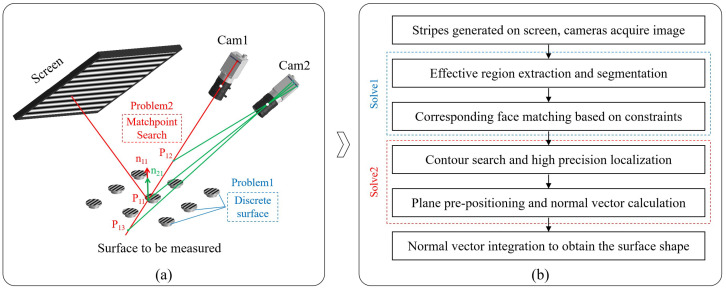
Measurement system structure and algorithm flow. (**a**) System structure diagram. (**b**) Algorithm flow.

As illustrated in Figure 1a, the measurement system comprises two cameras and a screen. The light emitted from the screen’s pixels is reflected by the surface and then captured by the cameras. By calculating the directions of the incident and reflected light rays, we can determine the surface’s normal vector. Ultimately, the 3D global shape of the target surface is reconstructed by integrating the slope data. Based on the research findings, the current reconstruction algorithms primarily encounter two main issues, as depicted in Figure 1a. First, the surfaces to be measured are discrete from one another, making it challenging to directly integrate multiple surface types. Second, the binocular system’s measurement accuracy is high, along with excellent integration capabilities. However, for each valid camera sampling point, it necessitates a search along the light direction within a specific area (from $P_{12}$ to $P_{13}$) until the surface normal vectors ($n_{11}$ and $n_{21}$) computed by the two cameras align in the same direction. This search process is time-consuming and hampers the efficiency of the measurement system.

To achieve the batch measurement of specular planes and address the aforementioned challenges, the algorithm’s flow, as outlined in our manuscript, is depicted in Figure 1b. In the initial step, fringes of varying frequencies and periods are generated on the screen. The cameras perform image acquisition, and distortion correction is applied to the images. To ensure the stability of the measurement, the correspondence between camera pixels and screen pixels is established by using the four-step phase-shifting and multi-frequency (hierarchical) methods to achieve phase measurement and unwrapping [20]. Subsequently, in the second step, the effective region of the image is extracted based on the variation in fringes and segmented according to region connectivity. Moving on to the third step, corresponding surface matches are obtained based on the polar lines and normal vector constraints, thereby identifying the regions that correspond to the two cameras. In the fourth step, for each surface to be solved, the accuracy of contour extraction is further enhanced by searching within the vicinity of the polar lines. In the fifth step, the spatial position of the surface to be measured is determined based on the profile, and a plane pre-positioning method is employed to calculate the normal vector direction of the surface to be measured. Ultimately, in the final step, the shape of each plane to be measured is obtained via separate integration.

### 2.2. Discrete Surface Separation and Matching

PMD faces several challenges when applied to batch surface measurements. Firstly, the surfaces to be measured are discrete, making direct integration impractical. Consequently, it becomes necessary to segment the regions and perform separate integration to calculate the surfaces. Secondly, the inherent similarity of the surfaces in the images complicates the task of determining the corresponding positions in two cameras after segmentation by using direct feature matching. To address these issues, a matching method based on polar lines and normal vector constraints is introduced. This method consists of two key steps: first, the extraction of the effective measurement region, and second, the matching of surfaces based on the proposed constraints.

**Figure 2:**
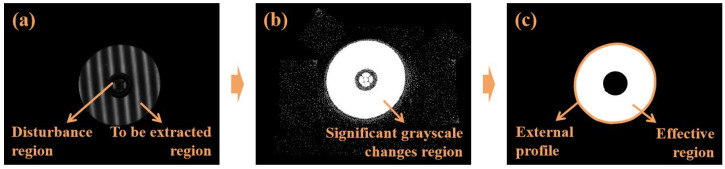
Effective-region and contour extraction using fringe contrast. (**a**) Specular fringe image of the surface to be measured. (**b**) Contrast calculation results (the higher the contrast, the larger the grayscale values). (**c**) Effective-region selection and contour extraction.

Throughout the measurement process, fringes of varying frequencies and periods are generated on the screen, and the light reflected from the screen’s surface is captured by the cameras. These captured images are subsequently employed to establish correspondence between the camera’s pixels and the screen’s pixels through phase unwrapping. Additionally, based on the grayscale variation in the acquired strip images, it is possible to extract the effective regions. As shown in Figure 2a, using one specular surface as an example, within the effective region where fringes are reflected, significant variations between different phase images acquired by the camera become readily apparent. Four images with a continuous change in fringes in the same period are extracted, assuming that their grayscale images are $I_{1}$, $I_{2}$, $I_{3}$, and $I_{4}$. According to the four-step phase-shifting principle [20], $I_{i}$ can be represented by Equation (1), where *A* represents the background grayscale, *B* denotes the modulation amplitude, and φ denotes the corresponding phase. The average grayscale ($I_{avg}$) and the grayscale variation in each pixel ($I_{chg}$) can be computed according to Equations (2) and (3). The region with high variation is shown in Figure 2b. Furthermore, the determination of the effective region can be carried out by using a threshold value. By substituting Equations (1) and (2) into Equation (3), the range of $I_{chg}$ can be calculated by using Equation (4). The calculation of the *B* value can be obtained from Equation (5). Clearly, the threshold can be determined based on the value of *B*. Initially, the threshold’s initial value ($T_{0}$) can be calculated by using the multitude calculated from *B* in the effective numerical region. By considering factors such as noise and the distance of the surfaces, in order to include all surfaces in the measurement, we scale down $T_{0}$ by a coefficient (*k*) of less than 1 to obtain the filtering threshold (*T*), as shown in Equation (6). The regions with $I_{chg}$ values greater than the threshold are selected, and finally, the desired effective region can be obtained within the area of the connected domain. Finally, the outer edge contour of the region is extracted, and the obtained region and contour are shown in Figure 2c.

(1) $$\begin{array}{c} I_{i}=A+B\cos\left(\varphi +i\cdot \pi /2\right) \end{array}$$

(2) $$\begin{array}{c} I_{avg}=\left(I_{1}+I_{2}+I_{3}+I_{4}\right)/4 \end{array}$$

(3) $$\begin{array}{c} I_{chg}=\left(\right|I_{1}-I_{avg}\left|+\right|I_{2}-I_{avg}\left|+\right|I_{3}-I_{avg}\left|+\right|I_{4}-I_{avg}\left|\right)/4 \end{array}$$

(4) $$\begin{array}{c} I_{chg}=B\cdot \left(|\sin\varphi \right|+\left|\cos\varphi |\right)/2\geq B/2 \end{array}$$

(5) $$\begin{array}{c} B=\frac{\sqrt{{\left(I_{1}-I_{3}\right)}^{2}+{\left(I_{2}-I_{4}\right)}^{2}}}{2} \end{array}$$

(6) $$\begin{array}{c} T=T_{0}/2\cdot k \end{array}$$

Through the method mentioned above, effective-area segmentation and contour extraction can be achieved. It is further necessary to confirm the corresponding matching relationship between the images acquired by the two cameras without features. The matching method based on polar lines and normal vector constraints can effectively solve this problem. Taking camera 1 as the benchmark, at any sampling point on the extracted contour, a polar line corresponding to the point in the image captured by camera 2 can be obtained. The equation of the polar line can be calculated by using Equation (7). In the equation, coordinate $u_{1}$, $v_{1}$ represents the pixel coordinates of the selected point in camera 1. The parameters $u_{x}$ and $v_{y}$ represent the *u* and *v* coordinates of the polar line point in camera 2, which represents the straight-line equation. The parameters $k_{1}$, $k_{2}$, *r*, and *t* represent the intrinsic and extrinsic parameters of cameras 1 and 2 and their relative spatial relationships (rotation and translation), respectively, which can be obtained through camera calibration [21].

(7) $$\begin{array}{c} \left(u_{x},v_{y},1\right)\cdot \left\{k_{2}^{-T}\cdot \left\{t^{\times }\left[r\cdot k_{1}^{-1}{\left(u_{1},v_{1},1\right)}^{T}\right]\right\}\right\}=0 \end{array}$$

**Figure 3:**
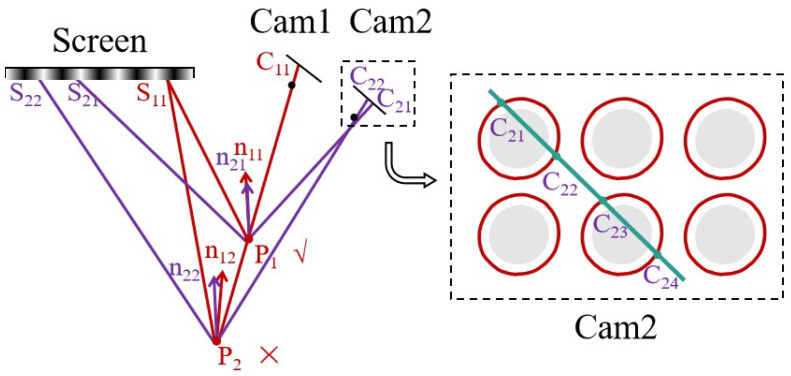
Selection of correspondence points based on polar lines and normal vector constraints.

As shown in Figure 3, the polar line may have several intersection points ($C_{21}$, $C_{22}$, …$C_{24}$) with respect to the extracted contour in camera 2. Since the surfaces of the specular reflection lack features and are similar, directly determining the corresponding points through features is difficult. Therefore, further determining the corresponding points through the difference in normal vectors of the intersection points is necessary. Taking $C_{21}$ and $C_{22}$ as an example, according to the principle of stereo vision, a corresponding pair of points on cameras 1 and 2 can determine a point in space, based on pixel coordinates and camera parameters. Thus, different points in camera 2 can determine a spatial corresponding point ($P_{1}$, $P_{2}$) with the point selected in camera 1 (assumed to be $C_{11}$). After determining the spatial position of the point, its corresponding surface normal vector n11 can be calculated according to Equation (8). The parameter $S_{11}P_{1}$ represents the vector from the light emitted by the screen to reflection point $P_{1}$, and $P_{1}C_{11}$ represents the direction vector of the light from the reflection point to the camera. The calculation method of $n_{12}$ is the same. Since the normal vector directions calculated in the left and right cameras for the same point on the object surface should be the same, the point with the smallest difference in the normal vector directions of the left and right cameras calculated via $P_{1}$ and $P_{2}$ is the corresponding point of the edge contour in the two cameras (as there will be deviations in the actual solution and the difference is not exactly equal to 0, the smallest difference is used as the basis for judgment). The same principle applies when judging multiple intersection points. Obviously, the area where the corresponding point is located is the corresponding area of the selected area in camera 1.

(8) $$\begin{array}{c} n_{11}=\frac{\vec{P_{1}C_{11}}-\vec{S_{11}P_{1}}}{\left|\right|\vec{P_{1}C_{11}}-\vec{S_{11}P_{1}}\left|\right|} \end{array}$$

Through the above steps, the automatic segmentation and corresponding area matching of batch surfaces can be performed, and the problems of discontinuous surfaces and a lack of surface features can be solved. Subsequently, the surface calculation algorithm is further used to perform the batch measurement of specular planes.

### 2.3. Rapid Normal Vector Computation

#### 2.3.1. Principles of Methodology

The process of matching and searching between the two cameras is very time-consuming. To solve this problem, we propose a fast reconstruction method based on plane pre-positioning. Since the surface to be measured is a specular plane with little undulation, this method can improve the solution efficiency while ensuring high measurement accuracy. It mainly consists of two steps. The first step is a high-precision search for the contour of the surface to be measured. The second step is spatial plane fitting and surface normal vector calculation based on contour positioning.

**Figure 4:**
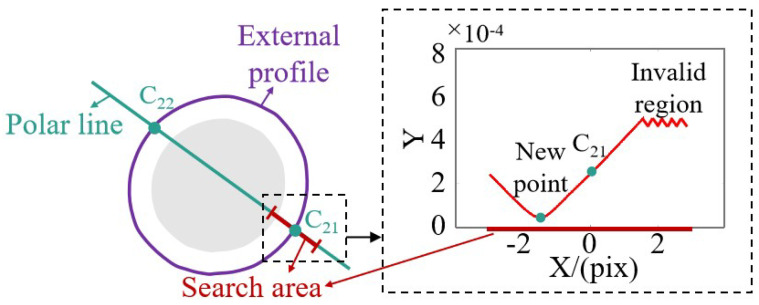
High-precision contour correspondence point calculation.

In Section 2.2, we have completed the extraction of the contour and the judgment of the corresponding points based on polar lines and normal vector constraints. However, in the actual contour extraction process, due to the fact that the image may need to be dilated and eroded, contour extraction may exhibit poor accuracy. If only the intersection point of the polar line and the contour in the image of camera 2 is used as the corresponding point of the image selection point in camera 1, there will be a certain degree of deviation, which may even be a few pixels. This deviation has no effect on the judgement of the correspondence point, because despite the deviation, the difference between normal vectors for the same correspondence point is still at the minimum. However, for the plane pre-positioning process, if we only rely on the coarsely extracted contour for the correspondence point calculation and plane fitting, it will introduce a large deviation with respect to the plane’s position. Therefore, in order to reduce the influence of contour extraction accuracy on plane pre-positioning, as shown in Figure 4, a small area near the corresponding point, $C_{21}$, of the contour extracted by camera 2 (within the effective area where the phase change can be extracted) is searched along the polar line. The choice of the region is not unique and can be adjusted based on practical considerations. Opting for a region that is too small may result in the inability to locate the minimum point, while selecting a region that is too large may lead to an extended search time. The search range chosen in this paper extends in two directions along the polar line, with the initial point as the center and spanning 3 pixels in each direction. The two-camera normal vector difference (the mode of the normalized difference) for each point in the search area is also plotted in Figure 4, with the horizontal axis indicating the relative position to the initial point and the vertical axis indicating the difference in normal vectors computed by the two cameras. Such an approach can significantly improve the corresponding contour point’s extraction accuracy and make up for the loss of accuracy during extraction.

**Figure 5:**
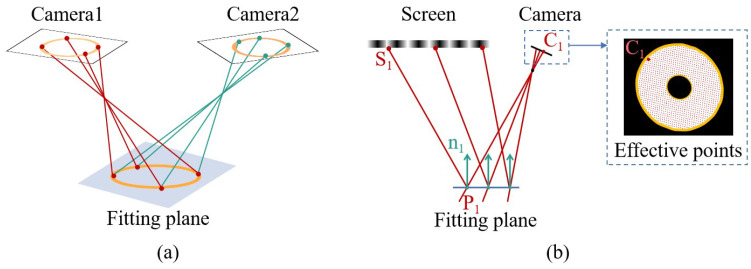
Normal vector calculation using plane pre-positioning. (**a**) Contour positioning and plane fitting. (**b**) Calculation of surface normal vectors using plane pre-positioning.

Furthermore, as illustrated in Figure 5a, the spatial location of the edge of the actual surface to be measured can be determined based on the corresponding points on the contours of the two camera images. Since the surfaces to be measured are planes, the least squares method can be applied to fit a plane to the spatially positioned contour. This plane serves as the position of the surface to be measured. Following the determination of the spatial plane for the surface, as shown in Figure 5b, any camera can be selected, and the intersection points of the corresponding rays from all pixels within its effective area with the fitted plane are computed. These intersections represent the spatial positions corresponding to the pixel points. Upon establishing the spatial position of the point, the directions of the incident and reflected light rays in space can be calculated based on the spatial positions of the corresponding camera pixel and screen pixel. Subsequently, the surface’s normal vector can be further computed for the entire surface to be measured.

The plane pre-positioning method only involves small-scale searches for corresponding points on the image contour, followed by a straightforward calculation of plane intersections for each valid pixel point. This approach eliminates the necessity to search for corresponding points in another camera for every valid pixel, leading to a substantial enhancement in algorithm efficiency.

#### 2.3.2. Accuracy Validation of the Algorithm

In our proposed algorithm, since the surface to be measured will have some undulations, only using the intersection point of the fitted plane and the light ray as the location of the point to be measured will introduce some pre-positioning deviations with respect to the point being measured. In this section, we will evaluate the impact of this algorithm on measurement accuracy through simulations.

**Figure 6:**
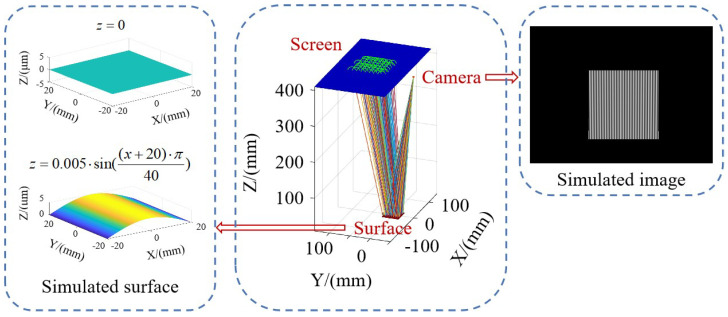
Diagram of the system imaging simulation mode.

The simulation of the measurement system was implemented by using Matlab programming. The simulation model, illustrated in Figure 6, employs inverse simulation by tracing light rays backward from the camera pixels to the screen in order to generate fringe images captured by the simulated camera. It calculates the intersection point of each light ray with the known surface (provided as simulation input) and sequentially computes the surface’s normal vector and shape. The simulation involves a comparison of two algorithms for positioning the surface. These algorithms are referred to as Algorithm 1 (an approximation based on the results of the two-camera matching search) and Algorithm 2 (replacing the surface with a contour-fitting plane). The simulated surfaces for measurement include a flat surface and a sinusoidal surface with a 5 μm amplitude. Additionally, to simulate instances of inaccurate plane fitting, the contour-fitting plane is vertically shifted by 5 μm along the normal vector direction.

**Figure 7:**
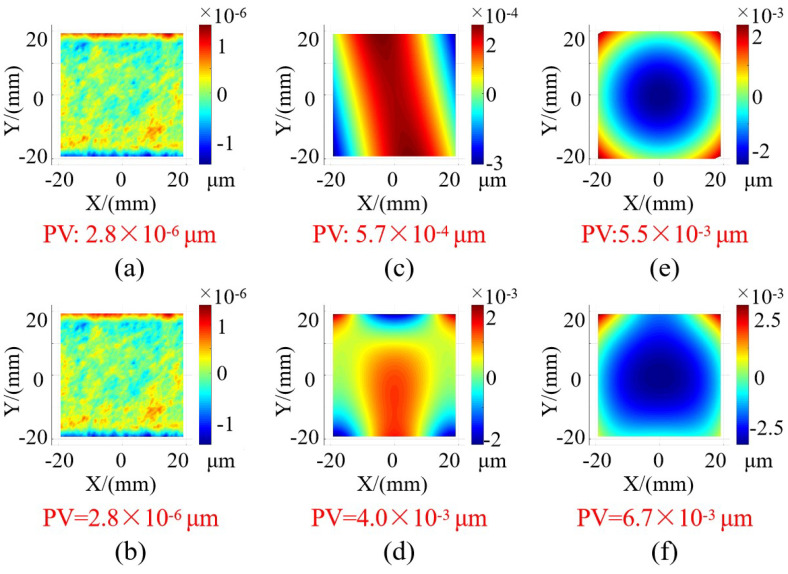
Deviation of simulation results. (**a**) Simulation reconstruction of plane using Algorithm 1. (**b**) Simulation reconstruction of plane using Algorithm 2. (**c**) Simulation reconstruction of sinusoidal surface using Algorithm 1. (**d**) Simulation reconstruction of sinusoidal surface using Algorithm 2. (**e**) Simulation reconstruction of plane using Algorithm 1 with 5 μm positioning deviation. (**f**) Simulation reconstruction of the sinusoidal surface using Algorithm 1 with 5 μm positioning deviation.

The simulation results are depicted in Figure 7, illustrating the disparity between the simulated reconstruction outcomes and the ideal surface shape. The peak-to-valley (PV) value of this difference indicated in Figure 7a,b indicates that the two measurements are identical when dealing with an ideal plane. As shown in Figure 7c,d, the utilization of plane pre-positioning introduces a slight measurement deviation compared with the accurately positioned points to be measured. However, for a surface undulation of 5 μm, this measurement deviation remains below 5 nm, which equates to an effect on accuracy of roughly one part in a thousand and can be considered negligible during the measurement process. Figure 7e,f simulate the impact of contour positioning deviation by deliberately shifting the fitting plane of the surface to be measured by 5 μm along the vertical direction. It is evident that this 5 μm positioning deviation results in a deviation below 10 nm, indicating a relatively minor influence of plane positioning deviation on the measurement results.

Simulation results confirm that the positioning deviation of the point to be measured introduced by the plane pre-positioning has a minimal impact on the measurement outcome. Our algorithm ensures high measurement accuracy when applied to planar measurement objects.

## 3. Experiment

### 3.1. Measurement System

#### 3.1.1. System Construction

**Figure 8:**
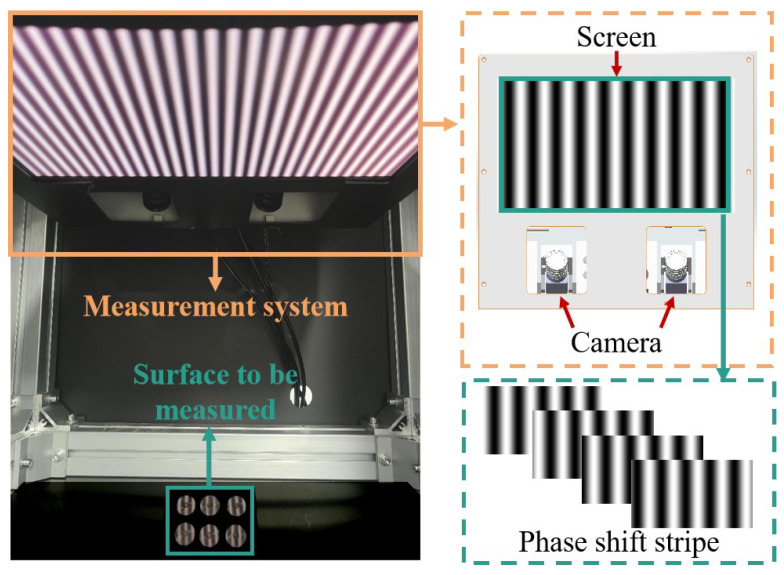
Picture of measurement system.

The image of the measurement system is illustrated in Figure 8, consisting of a screen, dual cameras, and their respective lenses. The system’s parameters are detailed in Table 1. The measurement process involves projecting fringes of varying frequencies and periods onto the screen (after gamma correction), while the cameras capture the reflected fringes from the target surface.

#### 3.1.2. System Calibration

**Figure 9:**
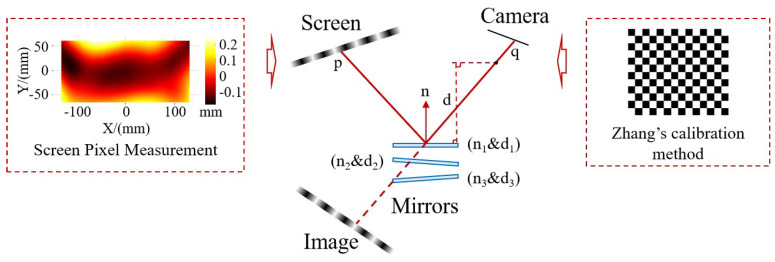
A schematic diagram of the calibration method of the system.

The key components of the PMD system include the camera and the screen. The camera is responsible for determining the direction of the reflected light from the surface under investigation, while the screen determines the direction of the incident light. Consequently, a calibration process is essential before conducting measurements. The calibration procedure and method are outlined in Figure 9, encompassing the calibration of the intrinsic and extrinsic parameters of the cameras, screen pixel calibration, and the calibration of the relative spatial positions of the screen and the camera. For the intrinsic parameters of cameras and the relative spatial relationship, we employed Zhang’s camera calibration method [21]. The screen’s pixel points are meticulously measured by using binocular vision to establish the spatial position of each pixel under the screen’s coordinate system. Finally, a calibration approach based on plane mirrors is utilized to align the camera’s coordinate system with the screen’s coordinate system [22]. Via this method, the camera captures the screen’s mirrored image from three distinct positions of the plane mirror. Each shot generates a set of rotational translation matrices, relating the screen’s mirror coordinate system to the camera’s coordinate system. Leveraging the plane mirror’s normal vector perpendicular to the mirror’s surface, along with the distance between various plane mirrors and the coordinate origin, a least squares matrix is constructed. This matrix facilitates the determination of the screen’s position relative to the camera. After calibration, the system can guarantee time stability. Furthermore, to maintain measurement accuracy, we routinely apply calibration corrections to the system.

### 3.2. Accuracy and Repeatability Validation

#### 3.2.1. Accuracy Validation

**Figure 10:**
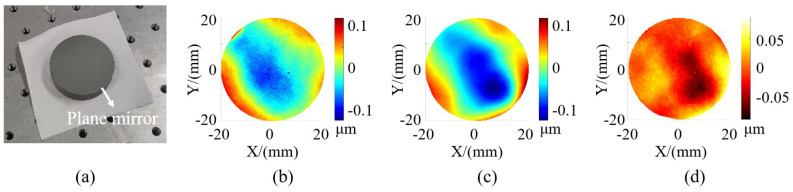
Picture of plane mirror and measurement results. (**a**) Picture of plane mirror. (**b**) Measurement results of interferometer. (**c**) Measurement results of PMD (our method). (**d**) Measurement deviation.

To assess the accuracy of the system, we measured a high-precision circular plane mirror with a 40 mm diameter, as displayed in Figure 10a. This plane mirror was subjected to measurements by using both an interferometer and the measurement system we proposed. The measurement results are presented in Figure 10, with Figure 10b depicting the result of the interferometer and Figure 10c showcasing the result of our system of the plane mirror. From the figure, we can see that the measurement results of our method are close to the measurement results of the interferometer. For a clearer comparison, we calculated the deviation between the measurement results obtained from two measurement methods, and the measurement deviation is shown in Figure 10d. (The measurement result was slightly translated along the Z-direction to ensure symmetrical deviation.) The measured deviation ranged within ±0.092 μm, demonstrating that our system possesses high measurement accuracy. For quantitative analyses, Table 2 provides quantitative evaluation error indices for the plane mirrors measured by using both methods. The table includes peak-to-valley (PV) and root mean square error (RMS) values. Notably, several quantitative indices exhibit proximity to each other.

#### 3.2.2. Repeatability Validation

**Figure 11:**
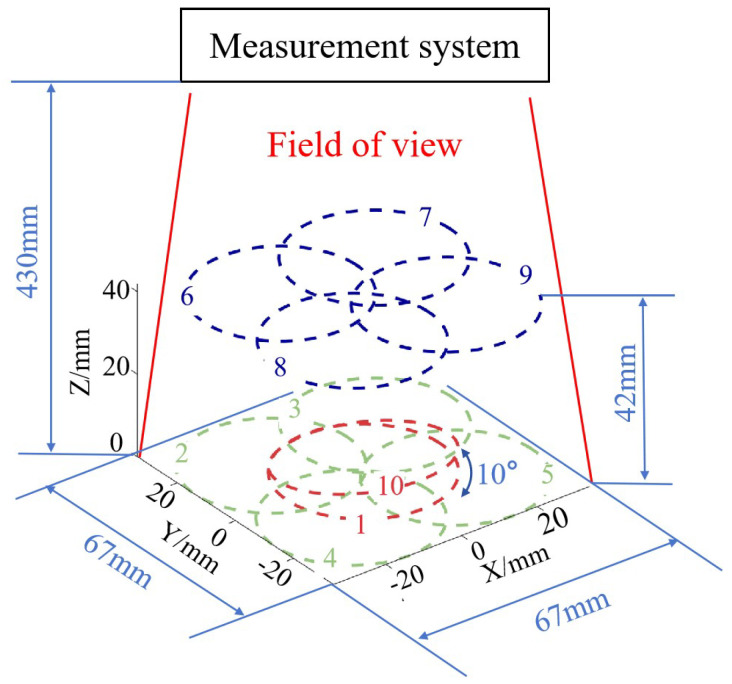
Schematic of plane mirror positions in repeatability measurements.

**Figure 12:**
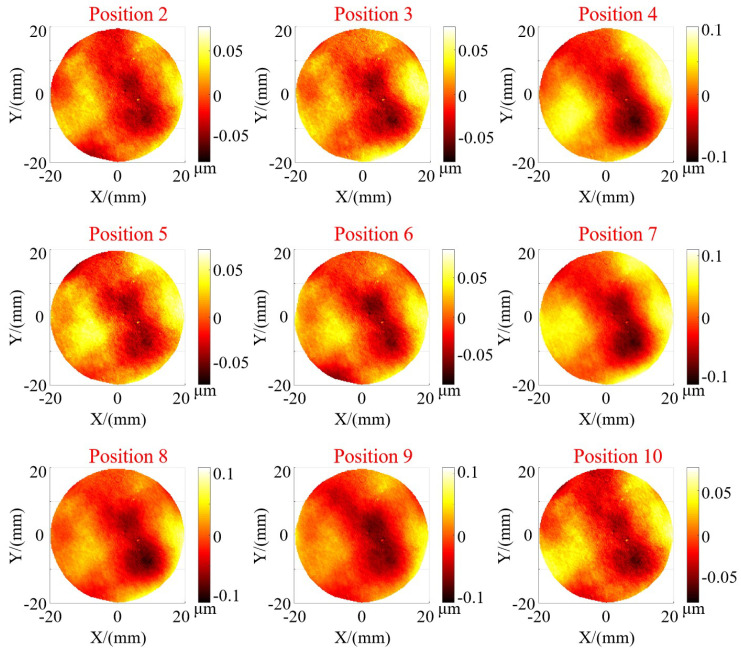
Measurement deviation for plane mirror in different positions.

To accomplish batch measurements, it is imperative to ensure that the system maintains stable measurement capabilities across all positions within the field of view. To assess the repeatability of the measurement system and its measurement capabilities at different positions within the field of view, as shown in Figure 11, the plane mirror underwent translation and rotation within a certain spatial range to validate the measurement accuracy at different heights, positions, and rotation angles. A total of 10 measurement experiments were conducted, with a height variation of approximately 42 mm and an angle variation of about 10°. The positions were distributed across the entire field of view at different heights. We calculated the deviation between the results of ten measurements and those of the interferometer. The measurement results for position 1 are presented in Figure 10d, while the remaining results are shown in Figure 12. Firstly, from the deviation figure, it can be observed that the results of the ten measurements exhibit good repeatability. Moreover, we calculated the PV and RMS values of the deviation results, as shown in Table 3. The deviations from the ten measurements have similar PV and RMS values, with calculated standard deviation (SD) values of 0.0314 μm and 0.0049 μm, respectively. Certainly, the small SD value is somewhat related to the inherently small RMS value of the plane mirror itself. Based on these experimental results, it can be demonstrated that the system exhibits high measurement accuracy and stable results for the surface in different poses.

### 3.3. Batch Surface Measurement Experiments

#### 3.3.1. Batch Measurement and Automatic Evaluation Experiment

**Figure 13:**
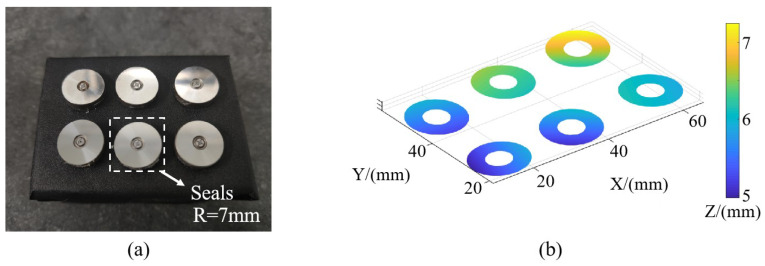
Picture of seals to be measured and measurement results. (**a**) Picture of seals to be measured. (**b**) Relative position measurement results of seals.

To demonstrate the capability of our method to simultaneously measure multiple specular surfaces, as illustrated in Figure 13a, we projected fringes onto multiple injector-sealing surfaces, capturing images for measurement. By utilizing the surface separation and matching method, along with the surface reconstruction algorithm presented in this paper, we acquired the profiles of multiple surfaces within a single measurement process. The spatial locations of these surfaces are depicted in Figure 13b, highlighting that several surfaces are not perfectly aligned within the same plane, and they are not entirely parallel to one another. This further underscores the advantage of our measurement method over interferometry. Specifically, it eliminates the need for precise surface leveling and positioning, greatly enhancing measurement efficiency.

**Figure 14:**
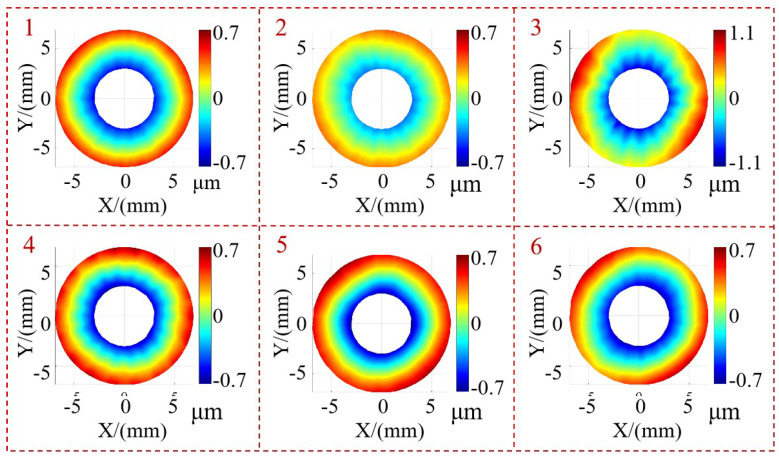
Measurement results for each surface to be measured.

Additionally, as we can individually compute the surface shape for each measured surface, we can easily perform automatic de-tilting and center translation for each face. The de-tilting method involves fitting a plane and rotating its normal vector to align with the Z-axis direction. The center adjustment involves translating the center of the measured surface to the origin of the coordinates. The center can be chosen as either the centroid or geometric center of the surface. The processed results are shown in Figure 14. Then, each surface can be assessed for qualification or further processing as per specific requirements. For instance, among the six measured surfaces, the PV value for surface 3 surpasses the acceptable range (2 μm), indicating a failed product requiring correction. The remaining inspected surfaces meet the criteria for qualification.

#### 3.3.2. Validation of Algorithm’s Robustness

**Figure 15:**
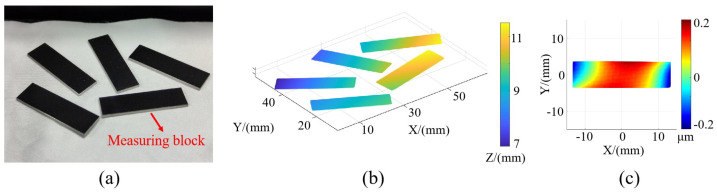
Picture of gauge blocks and measurement results. (**a**) Picture of gauge blocks. (**b**) Relative position measurement results. (**c**) Measurement results for a single gauge block.

Several gauge block surfaces were subjected to measurement by using the method presented in this study. As depicted in Figure 15a, the blocks were arranged in random positions, ensuring that they could reflect the screen fringes relative to the camera. The relative positions of the blocks and the shapes of the surfaces obtained through the algorithm outlined in this paper are displayed in Figure 15b,c. This experiment serves to illustrate the robustness of the proposed algorithm, which can successfully achieve surface separation, matching, and measurement even when the surfaces are randomly arranged and the surface profiles are varied.

### 3.4. Comparison of Performance Improvement

**Figure 16:**
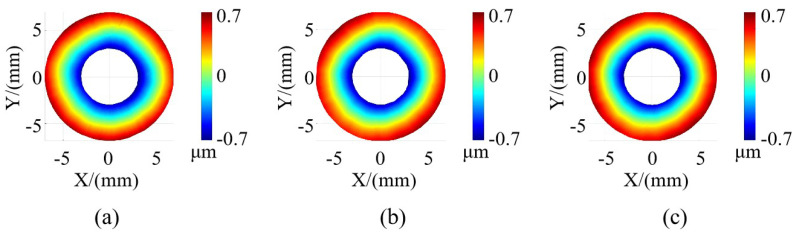
Measurement results for a single seal. (**a**) Reconstruction via the traditional match-based method. (**b**) Reconstruction via our method. (**c**) Measurement results of the interferometer.

To demonstrate the enhanced efficiency of the plane pre-positioning method proposed in this paper compared with the traditional matching-based method [8] and to ensure measurement accuracy even when the surface is not an ideal plane, both methods were employed to reconstruct a single-seal surface. The accuracy and efficiency of these reconstructions were compared. The measurement results are displayed in Figure 16. Furthermore, PV and RMS values for their respective surface shapes were calculated, and they are provided in Table 4. Notably, these diverse approaches yield similar measurement results, further validating the high measurement accuracy of the proposed method.

On the other hand, we conducted a comparison of the running times of the two solving methods. By using identical computing equipment and software (an Intel (R) Core (TM) i5-8300H CPU (Intel, Santa Clara, CA, USA) and Matlab 2017), we measured the time required to solve a single surface shape for both methods. The results clearly demonstrate that the plane pre-positioning method proposed in this paper reduces the solving time to only 20% compared with the matching-based reconstruction method, resulting in a substantial improvement in solving efficiency.

## 4. Conclusions

In addressing the challenge of rapid and high-precision batch flatness measurements, this paper introduces improvements to the traditional PMD method. We employ surface separation and corresponding surface matching to achieve discrete surface reconstruction. We effectively resolve the efficiency limitations of traditional binocular deflectometry systems by utilizing a plane pre-positioning approach, significantly enhancing the speed of surface reconstruction. The experimental results demonstrate that the proposed method allows for the measurement of batch specular plane flatness while maintaining high measurement precision. Moreover, it substantially reduces reconstruction times compared with existing algorithms. In the future, the relative position measurement of the surface can be investigated through methods such as contour positioning to achieve simultaneous high-precision measurement of surface profile and position.

## Figures and Tables

**Table 1:** **System parameters**.

Hardware	Parameters
Screen	Pixel size (0.16 mm)
Camera	Resolution rate (2048 × 1536)
Lens	Focal length (25 mm)

**Table 2:** **Plane mirror measurement results**.

	Interferometer	Our Method
PV (μm)	0.2339	0.2726
RMS (μm)	0.0398	0.0510

**Table 3:** **PV and RMS calculations of measurement deviations**.

Number	1	2	3	4	5	6
PV (μm)	0.1843	0.1552	0.1524	0.2072	0.1431	0.1710
RMS (μm)	0.0315	0.0225	0.0237	0.0354	0.0259	0.0253
Number	7	8	9	10	Avg	SD
PV (μm)	0.2247	0.2173	0.2198	0.1522	0.1824	0.0314
RMS (μm)	0.0362	0.0296	0.0325	0.0264	0.0289	0.0049

**Table 4:** **Single-seal measurement results and reconstruction time**.

	Match-Based Method [8]	Our Method	Interferometer
PV (μm)	1.3494	1.3537	1.3512
RMS (μm)	0.3645	0.3534	0.3592
Time (s)	11.93	2.16	

## Data Availability

Data are contained within the article.

## References

[1] Liu Y., Dai Y.C., Shen X.Y., Li D.S. (2020). Sealing test of gas valve cover of gas meter based on line laser triangulation method. Am. J. Opt. Photonics.

[2] Nguyen M.T., Lee J., Ghim Y.S., Rhee H.G. (2022). Real-time 3D measurement of freeform surfaces by dynamic deflectometry based on diagonal spatial carrier-frequency pattern projection. Measurement.

[3] Zhuang Y., Zheng Y., Lin S., Wang D., Zhang Y., Huang L. (2022). Surface shape distortion online measurement method for compact laser cavities based on phase measuring deflectometry. Photonics.

[4] Lakota S., Görög A. (2011). Flatness measurement by multi-point methods and by scanning methods. Ad Alta J. Interdiscip. Res..

[5] Yang S., Zhang G. (2018). A review of interferometry for geometric measurement. Meas. Sci. Technol..

[6] Burke J., Pak A., Höfer S., Ziebarth M., Roschani M., Beyerer J. (2023). Deflectometry for specular surfaces: An overview. Adv. Opt. Technol..

[7] Bothe T., Li W., von Kopylow C., Juptner W.P. (2004). High-resolution 3D shape measurement on specular surfaces by fringe reflection. Opt. Metrol. Prod. Eng..

[8] Knauer M.C., Kaminski J., Hausler G. (2004). Phase measuring deflectometry: A new approach to measure specular free-form surfaces. Opt. Metrol. Prod. Eng..

[9] Xu Y., Gao F., Zhang Z., Jiang X. (2018). A holistic calibration method with iterative distortion compensation for stereo deflectometry. Opt. Lasers Eng..

[10] Han H., Wu S., Song Z., Gu F., Zhao J. (2021). 3D reconstruction of the specular surface using an iterative stereoscopic deflectometry method. Opt. Express.

[11] Wang R., Li D., Zhang X., Zheng W., Yu L., Ge R. (2021). Marker-free stitching deflectometry for three-dimensional measurement of the specular surface. Opt. Express.

[12] Zhang X., Li D., Wang R. (2023). Screen-monitored stitching deflectometry based on binocular stereo vision. Measurement.

[13] Wang R., Li D., Zhang X. (2021). Systematic error control for deflectometry with iterative reconstruction. Measurement.

[14] Liu Y., Huang S., Zhang Z., Gao N., Gao F., Jiang X. (2017). Full-field 3D shape measurement of discontinuous specular objects by direct phase measuring deflectometry. Sci. Rep..

[15] Wang Y., Xu Y., Zhang Z., Gao F., Jiang X. (2021). 3D measurement of structured specular surfaces using stereo direct phase measurement deflectometry. Machines.

[16] Chang C., Zhang Z., Gao N., Meng Z. (2020). Improved infrared phase measuring deflectometry method for the measurement of discontinuous specular objects. Opt. Lasers Eng..

[17] Gao F., Xu Y., Jiang X. (2022). Near optical coaxial phase measuring deflectometry for measuring structured specular surfaces. Opt. Express.

[18] Gao F., Xu Y., Jiang X. (2022). Stereo deflectometry based automatic segmentation technique for measuring structured specular surfaces. Opt. Lasers Eng..

[19] Zhang Z., Wang Y., Gao F., Xu Y., Jiang X. (2022). Enhancement of measurement accuracy of discontinuous specular objects with stereo vision deflectometer. Measurement.

[20] Zuo C., Huang L., Zhang M., Chen Q., Asundi A. (2016). Temporal phase unwrapping algorithms for fringe projection profilometry: A comparative review. Opt. Lasers Eng..

[21] Zhang Z. (2000). A flexible new technique for camera calibration. IEEE Trans. Pattern Anal. Mach. Intell..

[22] Xiao Y.L., Su X., Chen W. (2012). Flexible geometrical calibration for fringe-reflection 3D measurement. Opt. Lett..

